# Epidemiology and Characteristics of Invasive Yeast Infections in Patients with Hematologic Diseases: 12-Year Single-Center Retrospective Cohort Study

**DOI:** 10.3390/jof11080585

**Published:** 2025-08-08

**Authors:** Dong Young Kim, Keon Oh, Minseung Song, Hyemin Kweon, Dukhee Nho, Hanter Hong, Raeseok Lee, Dong-Gun Lee, Sung-Yeon Cho

**Affiliations:** 1 Vaccine Bio Research Institute, College of Medicine, The Catholic University of Korea, Seoul 06591, Republic of Koreasymonlee@catholic.ac.kr (D.-G.L.); 2 Division of Infectious Diseases, Department of Internal Medicine, Seoul St. Mary’s Hospital, College of Medicine, The Catholic University of Korea, Seoul 06591, Republic of Korea; 3 Catholic Hematology Hospital, The Catholic University of Korea, Seoul 06591, Republic of Korea

**Keywords:** candidemia, co-infection, *Cryptococcus*, incidence, invasive candidiasis, invasive fungal infections, mortality, yeast

## Abstract

Invasive yeast infections (IYIs) remain a significant cause of morbidity and mortality in patients with hematologic diseases. We retrospectively analyzed 193 IYI episodes among 179 patients admitted to a tertiary hematology hospital (2012–2023). *Candida* species accounted for 91.7% (n = 177), while non-*Candida* yeasts comprised 8.3% (n = 16). Among invasive candidiasis, non-*albicans Candida* spp. were predominant, representing 76.8% (136/177), with *C. tropicalis* (36.2%, 64/177) being the most frequently isolated species. Among non-*Candida* yeasts, *Cryptococcus neoformans* (n = 10) was the most commonly identified pathogen. The incidence and 42-day mortality rate of IYIs were 0.199 and 0.095 per 1000 patient-days, respectively. The 42-day case-fatality rate remained high at 47.7%. In categorical analysis, age >65 years, corticosteroid use, elevated lactate (>2 mmol/L), neutropenia (<500/mm^3^), vasopressor use, and mechanical ventilation were more common in non-survivors. Primary bloodstream infections were more frequent in non-survivors, whereas catheter-related and abdominal-origin infections were predominant among survivors. Concomitant bacteremia was observed in 32.6% of IYI cases (n = 63), with *Enterococcus faecium* being the most frequently isolated co-pathogen. Our findings illustrate the evolving epidemiology of IYIs in hematologic patients, marked by the emergence of *C. tropicalis* as the predominant species, sustained mortality, and frequent bacterial co-infections, collectively reflecting the substantial clinical burden of IYIs.

## 1. Introduction

Invasive fungal infections (IFIs) remain a major cause of morbidity and mortality in patients with hematologic malignancies due to chemotherapy-induced neutropenia, increasing use of novel agents in relapsed or refractory cases, and the frequent use of indwelling catheters [1,2]. Among IFIs, invasive yeast infections (IYIs), primarily invasive candidiasis, still account for a significant proportion of cases, although *Aspergillus* spp. infections are generally more common in patients with hematologic malignancies [3]. Despite appropriate treatment, invasive candidiasis carries a mortality rate of approximately 35% [4]. Over time, the epidemiology of IYIs has evolved, influenced by changing clinical practice and antifungal prophylaxis strategies.

*Candida albicans* has traditionally been the most prevalent species in invasive yeast infections [5]. However, recent studies have highlighted geographic and demographic variability in IYI, with a rising prevalence of non-*albicans Candida* species among patients with hematologic diseases, both globally and within Asia [6,7,8,9]. Notably, *C. glabrata* (or *Nakaseomyces glabratus*) is predominant in Northern American and Europe, whereas *C. tropicalis* or *C. parapsilosis* are more commonly observed in Asia [10]. In Korea, non-*albicans Candida* species have also become predominant, accounting for 60% of all candidemia cases in large tertiary centers [11,12].

In addition to *Candida* species, rare yeasts such as *Cryptococcus* spp. and *Trichosporon* spp. also warrant greater attention due to their associated mortality. The SEIFEM-2004 registry reported that the incidence of *Cryptococcus* spp. and *Trichosporon* spp. infections among patients with hematologic malignancies was 0.07% and 0.06%, respectively, compared to 1.5% for *Candida* spp. [13]. Although *Cryptococcus neoformans* has been more extensively studied in the context of HIV infection, it remains a clinically significant pathogen even among non-HIV-infected individuals, with reported mortality rates ranging from 8% to 20% [14].

With antifungal prophylaxis now widely used in high-risk hematology patients, including those undergoing hematopoietic stem cell transplantation (HSCT) or intensive chemotherapy, timely surveillance and detailed epidemiologic data are essential to guide clinical practice. Moreover, data on non-*Candida* yeast infections in patients with hematologic malignancies remain limited [15].

Therefore, this study aimed to characterize IYIs in patients with hematologic diseases by analyzing current trends in incidence and mortality, identifying species distribution and clinical features, including yeast–bacterial co-infections.

## 2. Materials and Methods

### 2.1. Study Design and Data Collection

A retrospective cohort study was conducted by reviewing electronic medical records of adult patients (≥18 years) with hematologic diseases who were admitted to the Catholic Hematology Hospital (CHH) of Seoul St. Mary’s Hospital between January 2012 and December 2023. CHH is one of the most active HSCT centers in Asia, with over 10,000 cumulative transplants performed since 1983, including 610 HSCTs in 2024. Demographic and clinical data such as age, sex, underlying diseases, laboratory and microbiologic results were collected.

### 2.2. Definitions

Only proven IYIs, defined according to the European Organization of Research and Treatment of Cancer/Mycoses Study Group Education and Research Consortium (EORTC/MSGERC) criteria [16], were included in the analysis. Corticosteroid usage was defined as therapeutic doses (≥0.3 mg/kg/day) for ≥3 weeks within 60 days preceding IYI diagnosis [16]. The origin of each IYI was determined based on pathogen identification from clinically relevant specimens and/or clinical or radiological evidence of infection in a specific organ, as assessed by consensus of at least two infectious disease specialists. Disseminated IYI was defined as the involvement of two or more non-contiguous organs with sterile specimens [17]. IYI episodes were considered to be separate episodes, if they were ≥30 days apart and preceded by resolution of symptoms and microbiological clearance of infection [18]. Concomitant bacteremia was defined as bacterial isolation from at least one blood culture within 3 days before or after IYI diagnosis. If the blood samples yielded potential skin contaminant isolates, these isolates were considered true pathogens only if antimicrobial treatment was initiated under the supervision of infectious disease specialists and if the patient presented with clinical signs and symptoms consistent with infection [19]. Treatment failure was defined as (1) death during antifungal therapy, (2) persistent isolation of the same species from blood or other sterile site specimens, or (3) minor or no improvement in attributable symptoms and signs of IYIs plus no improvement of radiological findings if additional cultures are not feasible [20]. Breakthrough IYI was defined as the occurrence of infection in patients who had received systemic antifungal agents for ≥7 days and in whom the IYI episode occurred while on treatment or within 72 h after discontinuation of antifungal therapy, regardless of the treatment intention (i.e., prophylactic, empiric, or pre-emptive) [21,22,23].

### 2.3. Statistical Analysis

Poisson regression was used to evaluate annual trends in incidence and mortality, and beta regression was used to assess changes in case fatality rate over time. The coefficient of determination (R^2^) was calculated for each model. Model diagnostics, including overdispersion checks and residual analysis, were conducted using the DHARMa package in the R packages (R 4.5.0), confirming acceptable model fit without evidence of overdispersion or systematic residual patterns. Clinical characteristics of IYIs were analyzed using categorical or continuous variables, stratified according to 42-day overall mortality status. Categorical variables were compared using the Chi-square test or Fisher’s exact test, and continuous variables were analyzed using Student’s *t*-test or Wilcoxon’s rank-sum test, as appropriate. A Cox regression analysis was performed to evaluate risk factors associated with mortality in IYIs. Variables with a *p*-value < 0.100 in the univariate analysis were included in the multivariate model. All statistical analyses were performed using R software (version 4.4.3), with two-sided *p*-values < 0.05 considered statistically significant.

## 3. Results

### 3.1. Incidence and Mortality of IYIs

**Figure 1:**
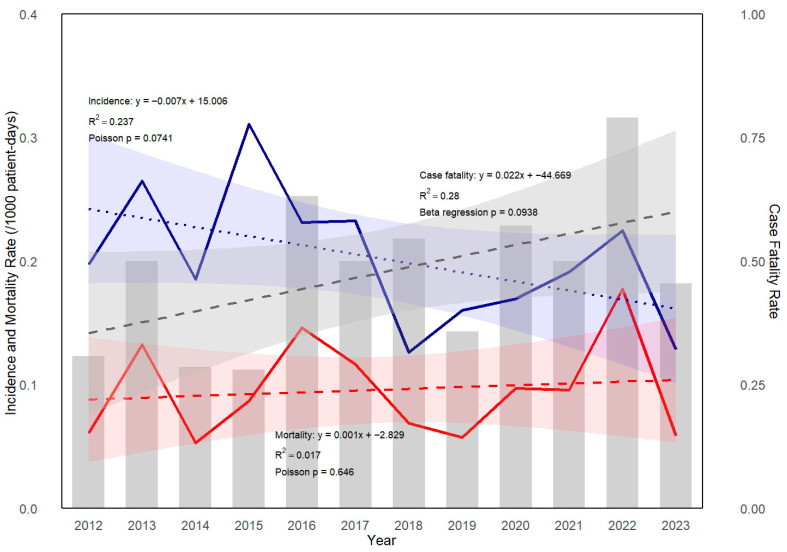
Trend of incidence, mortality, and case fatality rate of invasive yeast infection (IYI) in patients with hematologic diseases. Incidence (blue solid line) and mortality rate (red solid line) of IYI were analyzed using Poisson regression. A modest decreasing trend in incidence was observed over the years (*p* = 0.073, by Poisson regression). The case fatality rate (gray bars) demonstrated a non-significant upward trend (*p* = 0.095, by beta regression). Their trend lines estimated by regression are shown as dashed lines.

A total of 193 IYI episodes were identified in 179 patients, including 12 patients who experienced 2 distinct IYI episodes and 1 patient with 3 independent IYI episodes, as defined by our study criteria. Six cases involved mixed yeast infections, primarily involving multiple *Candida* spp. There was no case of a mixed *Candida* and non-*Candida* yeast spp. infection. The incidence and 42-day overall mortality rate for IYI were 0.199 and 0.095 cases per 1000 patient-days, respectively, during the study period. Although a decreasing trend was observed in the annual incidence, and the mortality rate of IYI tended to increase over time, none of these trends reached statistical significance (*p* = 0.073, *p* = 0.650 by Poisson regression; as shown in Figure 1. The 42-day overall case fatality rate from the time of IYI diagnosis was 47.7% throughout the study period. Although year-to-year variations were observed, the annual trend did not reach statistical significance (*p* = 0.095 by beta regression analysis). Breakthrough infections accounted for 19.2% (n = 37) of all IYI cases. Fluconazole was the most commonly used antifungal agent in breakthrough IYIs (31.5%), followed by posaconazole (19.3%), voriconazole (19.0%), and liposomal amphotericin B (16.7%). No clear trend of increasing or decreasing incidence of breakthrough infections was observed during the study period.

### 3.2. Species Distribution of IYIs

**Figure 2:**
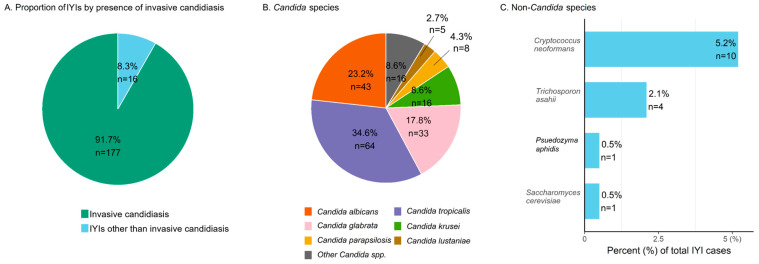
Species distribution of invasive yeast infections in patients with hematologic diseases. (**A**) Distribution of *Candida* spp. and non-*Candida* spp. infections. (**B**) Species distribution within *Candida* spp. “Other *Candida* spp.” includes *C. dubliniensis* (n = 2), *C. famata* (n = 1), *C. guilliermondii* (n = 1), *C. intermedia* (n = 1), *C. nivariensis* (n = 1), *C. norvegensis* (n = 1), *C. kefyr* (n = 1), *C. pelliculosa* (n = 1), *C. utilis* (n = 1), *Candida sp*. (n = 1), and only pathologic confirmation (n = 5). (**C**) Distribution of non-*Candida* spp. in IYI cases (percentages calculated based on the total number of IYI cases). Abbreviations. IYI, invasive yeast infection; spp., species.

Among all IYI cases, invasive candidiasis accounted for 91.7% (n = 177), and non-*Candida* IYIs for 8.3% (n = 16) (Figure 2A). Including isolates from mixed *Candida* spp. infections, a total of 185 *Candida* isolates were identified, and their species distribution is shown in Figure 2B. *C. tropicalis* was the most common spp. (34.6%, n = 64), followed by *C. albicans* (23.2%, n = 43) and *C. glabrata* (17.8%, n = 33). Non-*albicans Candida* species accounted for 76.8% (n = 142) of all *Candida* isolates. Non-*Candida* IYIs included *Cryptococcus neoformans* (n = 10), *Trichosporon asahii* (n = 4), *Pseudozyma aphidis* (n = 1), and *Saccharomyces cerevisiae* (n = 1). The 42-day overall mortality rate in non-*Candida* IYIs was 50.0% (8/16), with no significant difference compared to the invasive candidiasis group (47.5%, 84/177; *p* = 0.845). There was no statistically significant difference in 42-day overall mortality according to the causative organism of IYIs.

Antifungal susceptibility testing results were available in 114 cases (59.1%, 114/193), all of which were based on isolates obtained from blood cultures. Among these, 109 were *Candida* species, while 5 were non-*Candida* yeast species. For *Candida* isolates, without stratifying by species, fluconazole non-susceptibility was observed in 32.1% (35/109), and caspofungin non-susceptibility in 4.6% (5/109) of *Candida* spp. Among the non-*Candida* yeast species, susceptibility data were available for four *Cryptococcus neoformans* isolates, two of which were non-susceptible to both fluconazole and caspofungin. One *Trichosporon asahii* isolate exhibited caspofungin non-susceptibility.

### 3.3. Clinical Characteristics of IYIs

As shown in Table 1, the median age was 58 years (IQR 43–66). Among all patients, 28.5% (n = 55) were older than 65 years, and 54.9% (n = 106) were male. The most frequent hematologic diagnosis was lymphoma (22.3%, n = 43), followed by acute lymphoblastic leukemia (17.6%, n = 34), and both multiple myeloma and acute myeloid leukemia (14.5% each, n = 28). Neutropenia (absolute neutrophil count <500 /mm^3^) and prolonged neutropenia (lasting more than 3 weeks) preceded IYI development in 39.9% (n = 77) and 13.0% (n = 25) of cases, respectively.

Older age (≥65 years, 39.1% vs. 18.8%, *p* = 0.002), corticosteroid usage (35.9% vs. 20.8%, *p* = 0.020), and elevated serum lactate level (>2 mmoL/L, 25.0% vs. 4.0%, *p* < 0.001) showed higher prevalence in the deceased group. Also, both neutropenia (51.1% vs. 29.7%, *p* = 0.002) and prolonged neutropenia (19.6% vs. 6.9%, *p =* 0.009) at the time of IYI diagnosis were significantly more common in the deceased group. The use of vasopressors (66.3% vs. 8.9%, *p* < 0.001) and mechanical ventilation (33.7% vs. 6.9%, *p* < 0.001), both related to IYI, were associated with increased mortality.

The most common origin of IYI was primary bloodstream infection, accounting for 45.1% (n = 87) of cases, followed by catheter-related bloodstream infection (30.1%, n = 58), urinary tract (8.8%, n = 17), and abdominal sources (5.2%, n = 10). Other proven origins included the central nervous system (n = 4), skin and soft tissue (n = 2), pulmonary (n = 2), ruptured esophagus (n = 1), and skin (n = 1). When comparing survivors and non-survivors, primary bloodstream infection (55.4% vs. 35.6%, *p* = 0.006) was significantly more frequent among non-survivors, whereas catheter-related bloodstream infections (21.7% vs. 37.6%, *p* = 0.016) and infections related to abdominal origin (1.1% vs. 8.9%, *p* = 0.020) were more common in survivors. Among abdominal-origin IYI cases, 50.0% were hepatosplenic candidiasis (n = 5) and the remaining cases originated from the gastrointestinal tract (n = 5).

Treatment failure at 2 weeks after IYI diagnosis was observed in 47.2% (n = 91) of all cases. Approximately two-thirds (65.9%, 60/91) of treatment failures were due to death within 14 days of IYI onset. The remaining treatment failure cases were characterized by absent or delayed mycological clearance, with a median time to clearance of 21 days (range, 15–46 days) in the delayed mycologic clearance subgroup (n = 13).

In the survival analysis for 42-day overall case fatality using a Cox regression model, male sex, older age (>65 years), corticosteroid use, primary bloodstream infection, neutropenia, and elevated serum lactate level were associated with higher mortality. In contrast, catheter-related bloodstream infections were associated with a lower risk for 42-day mortality. In the multivariate Cox regression analysis, older age, corticosteroid usage, neutropenia, and elevated lactate level remained independently associated with increased mortality (in Appendix A).

### 3.4. Non-Candida IYIs

Among non-*Candida* IYIs, cryptococcosis was the most common, followed by trichosporonosis. Cryptococcal infections primarily presented as meningitis (40%, 4/10), followed by pulmonary cryptococcosis (20%, 2/10). Disseminated cryptococcosis was identified in only one case. Notably, two patients had positive blood cultures along with detectable cryptococcal antigenemia—an uncommon finding. Although invasive trichosporonosis is rare, it was associated with a high mortality rate of 75.0% (3/4).

### 3.5. Concomitant Bacteremia in IYIs

Concomitant bacteremia was identified in 63 IYI cases (32.6%), with a total of 79 bacterial isolates identified, detailed in Table 2. Polymicrobial bacteremia was observed in 14 cases—12 involving two bacterial species and two involving three. Gram-positive cocci were predominant (49/79, 62.0%). *Enterococcus faecium* was the most frequently isolated species (27/79, 34.2%), with 18 isolates (66.7%, 18/27) being vancomycin resistant. *E. faecalis* was identified in 4 cases (5.1%), all of which were sensitive to vancomycin. *Staphylococcus aureus* accounted for 3 cases (3.8%), and all isolates were methicillin sensitive. In contrast, 12 cases involved coagulase-negative *Staphylococcus* spp., of which 8 were methicillin-resistant. *Streptococcus* spp. were identified in 2 cases, and *Granulicatella adiacens* in one case. Gram-negative rods were found in 23 cases (29.1%), with *Klebsiella pneumoniae* being the most common (n = 9, 11.4%), followed by *Escherichia coli* (n = 5, 6.3%) and *Acinetobacter baumannii* (n = 4, 5.1%). In total, 80% of *E. coli* isolates (4/5) and 33.3% of *K. pneumoniae* isolates (3/9) were extended-spectrum β-lactamase (ESBL) producers. All *A. baumannii* isolates were carbapenem-resistant. *Stenotrophomonas maltophilia* and *Enterobacter cloacae* were each identified in two cases. Among the 63 IYI cases with concomitant bacteremia, bacterial pathogens were isolated concurrently with yeast from the same blood culture in 28.6% (n = 18) of cases.

## 4. Discussion

This study investigated the incidence and epidemiology of IYIs in patients with hematologic diseases. Invasive candidiasis accounted for the vast majority (91.7%) of IYI cases, while invasive non-*Candida* IYIs comprised approximately 8.3%. Notably, *C. albicans* was no longer the most common species; *C. tropicalis* emerged as the predominant isolate, accounting for about one-third of invasive candidiasis cases in patients with hematologic diseases. Breakthrough infections represented 19.2% of all IYI episodes, and yeast–bacterial co-infections were observed in 32.6% of cases. The 42-day overall mortality remained high at 47.7%. Although neutropenia remains an important risk factor, non-neutropenic patients now represent a substantial proportion of IYI cases (60.1%) in contemporary hematologic populations. While catheter-related yeast infections, which are potentially more controllable sources, were more common among 42-day survivors, primary bloodstream infections were more frequently observed in non-survivors. The presence of organ failure requiring vasopressors or mechanical ventilation was significantly more frequent in the non-survivor group.

The incidence of invasive candidiasis observed in this study is consistent with U.S. data showing a decline in hospital-acquired candidemia, which has been attributed to advances in bundled interventions for catheter care practices [24]. A reduction in incidence has also been linked to antifungal prophylaxis, particularly in high-risk hematologic patients receiving fluconazole or mold-active agents during periods of neutropenia [25]. A decline in *C. albicans* bloodstream infections has also been reported [26], along with a shift in species distribution from *C. albicans* to non-*albicans Candida* spp. over recent decades, particularly among patients with hematologic diseases [25]. Another multicenter surveillance study further reported that *C. tropicalis* was the most frequently isolated species (69%) in patients with hematologic malignancies, reflecting regional and population-specific trends in species distribution [27]. The incidence of cryptococcosis in our cohort was 1.03 cases per 100,000 patient-days. Although a direct comparison is difficult due to differences in calculation methods (percentage vs. patient-days), the SEIFEM-2004 study reported an incidence of 0.07% among patients with hematologic malignancies [13]. In studies of populations with human immunodeficiency virus (HIV), the estimated incidence was approximately 0.4–1.2 cases per 100,000 population in the United States [28]. While cryptococcal infections have traditionally received greater attention in the context of HIV infection, our findings highlight their occurrence in non-HIV immunocompromised populations as well.

Although the annual incidence of IYI in our study showed a decreasing trend and the mortality rate appeared to increase over time, neither trend reached statistical significance. This underscores the need for continued surveillance and further investigation into risk factors associated with IYI-related mortality.

In this study, 56.0% (108/193) of IYI episodes occurred in patients who were receiving conventional cytotoxic chemotherapy or had a history of allogeneic HSCT at the time of infection. Additionally, 13.5% (26/193) of cases occurred in patients receiving novel immunomodulatory agents, such as newer tyrosine kinase inhibitors and monoclonal antibodies, including 12 cases with refractory or relapsed disease following allogeneic HSCT. Patients with hematologic diseases generally possess multiple traditional risk factors for invasive candidiasis, including neutropenia, chemotherapy-induced mucosal barrier injury, frequent transfusions, broad-spectrum antibiotic use during febrile episodes, and the presence of indwelling catheters [2,29]. Although the current proportion of patients treated with novel agents remains modest, the emergence of IYIs in this group suggests that, beyond these traditional risk factors, immune modulation by targeted therapies may represent a new challenge in infection prevention [30].

Our findings also showed that patients with primary bloodstream infections had significantly higher 42-day overall mortality, whereas catheter-related bloodstream infections (CRBSIs) were associated with lower mortality. This difference may be explained by the fact that removal of the central venous catheter (CVC) in CRBSI can reduce treatment failure and mortality [31], and that appropriate abdominal source control—such as drainage of infected fluid collections, removal of infected tissue or foreign bodies, and correction of anatomical abnormalities—was implemented in our cases [32]. In our study, 75.9% of CRBSI cases (44/58) underwent CVC removal within 48 h of IYI confirmation. In contrast, in the group not classified as CRBSI, these patients had higher systemic inflammatory response syndrome (SIRS) scores and most of them had gut mucositis during nadir period related to intensive chemotherapy (median score 2.32 in the primary bloodstream infection group vs. 2.00 in the non-primary bloodstream group, *p* = 0.017 by Wilcoxon rank sum test). These findings may particularly explain the lower mortality risk observed in the CRBSI group.

It is also known that chronic disseminated candidiasis (CDC)–often referred to as hepatosplenic candidiasis in the recent literature–when not accompanied by concomitant bloodstream infections, is associated with a favorable prognosis [33], and no attributable deaths due to CDC were observed in our cohort.

Interestingly, we found that 32.6% (63/193) of IYI cases involved bacterial bloodstream co-infections in this study. Excluding one case of *Pseudozyma aphidis* infection, 62 cases represented concomitant *Candida*-bacterial bloodstream infections. Prior studies have reported mixed *Candida*-bacterial bloodstream infections in approximately 17–26% of patients, although the predominant bacterial species varied, including *K. pneumoniae*, *A. baumannii*, and *E. faecium* [34,35]. Consistent with these findings, *Enterococcus* spp., particularly *E. faecium*, were among the most frequently identified coinfecting organisms in our study. Notably, *E. faecium*, a common component of the altered intestinal microbiota, poses a particular clinical challenge due to its intrinsic and acquired resistance to multiple antibiotics, including vancomycin [36]. These *Candida*-bacterial co-infections may hold clinical relevance, particularly in immunocompromised patients with mucosal barrier injury or prolonged antibiotic exposure, as they may reflect translocation from the gastrointestinal tract due to altered microbiota, indicate underlying immune suppression, and carry prognostic implications [37,38]. In our cohort, however, there was no significant difference in the rate of co-infections between survivors and non-survivors, suggesting that the presence of bacterial co-infections alone may not directly influence short-term mortality. Such findings highlight the need to consider not only microbiota disruption but also interactions between co-infecting pathogens. These interactions, whether synergistic or antagonistic, may modulate the virulence and pathogenicity of both yeast and bacteria, and influence the host immune response [39]. Given these complex trans-kingdom dynamics, further research is warranted to elucidate the mechanisms underlying such co-infections and their prognostic implications.

This study stands out for focusing on IYIs in patients with hematologic diseases and for its inclusion of rare yeast pathogens, such as *Cryptococcus* spp. and *Trichosporon* spp., which are frequently excluded from prior research. However, this study has several limitations. First, the generalizability of the findings may be limited due to the single-center design, which does not capture institutional variability. Second, antifungal susceptibility data were available for only 59.1% of cases (114/193), and the heterogeneity of species and temporal variation precluded meaningful statistical analysis. Third, as the primary aim of this study was to describe the epidemiology of IYIs, it was not designed to assess factors influencing incidence, mortality, the occurrence of breakthrough IYIs, etc.

In conclusion, this study provides an updated characterization of IYIs in patients with hematologic diseases over the past 12 years. While the overall incidence of IYIs appeared to decline, the persistently high mortality rate underscores the clinical burden of these infections. The predominance of *C. tropicalis* over *C. albicans*, mixed yeast–bacterial bloodstream infections, and the emerging role of novel immunomodulatory agents suggest a shifting landscape in host–pathogen dynamics. As the population with hematologic diseases continues to evolve with increasingly complex treatment strategies, ongoing surveillance and individualized approaches to antifungal prophylaxis and therapy will be essential for optimizing patient care.

## Figures and Tables

**Table 1:** Clinical characteristics of invasive yeast infection cases in patients with hematologic diseases.

n, Median (IQR)	Total (n = 193)	42-Day Survival (n = 101)	42-Day Death (n = 92)	*p* Value
Age (years)	58 (43.0–66.0)	55 (41.0–64.0)	61 (51.0–69.0)	0.003
>65 years	55 (28.5%)	19 (18.8%)	36 (39.1%)	0.002
Sex (male)	106 (54.9%)	50 (49.5%)	56 (60.9%)	0.113
Hematologic diseases				0.773
Lymphoma	43 (22.3%)	21 (20.8%)	22 (23.9%)	
Acute lymphoblastic leukemia	34 (17.6%)	20 (19.8%)	14 (15.2%)	
Multiple myeloma	28 (14.5%)	17 (16.8%)	11 (12.0%)	
Acute myeloid leukemia	28 (14.5%)	14 (13.9%)	14 (15.2%)	
Myelodysplastic syndrome	22 (11.4%)	12 (11.9%)	10 (10.9%)	
Others *	38 (19.7%)	17 (16.8%)	21 (22.8%)	
Active hematologic diseases	134 (69.4%)	69 (68.3%)	65 (70.7%)	0.725
Cytotoxic chemotherapy	75 (38.9%)	39 (38.6%)	36 (39.1%)	0.941
History of allogenic HSCT	63 (32.6%)	36 (35.6%)	27 (29.3%)	0.351
Corticosteroid usage	54 (28.0%)	21 (20.8%)	33 (35.9%)	0.020
Immunosuppressant usage	38 (19.7%)	20 (19.8%)	18 (19.6%)	0.967
Hospital stay before IYI diagnosis (days)	15.0 (7.0–31.0)	15.0 (6.0–33.0)	17.0 (9.0–31.0)	0.361
Origin of Infection				0.003
Primary bloodstream infection	87 (45.1%)	36 (35.6%)	51 (55.4%)	0.006
CRBSI	58 (30.1%)	38 (37.6%)	20 (21.7%)	0.016
Urinary tract	17 (8.8%)	6 (5.9%)	11 (12.0%)	0.141
Abdomen ^†^	10 (5.2%)	9 (8.9%)	1 (1.1%)	0.020
Disseminated	11 (5.7%)	5 (5.0%)	6 (6.5%)	0.760
Others ^‡^	10 (5.2%)	7 (6.9%)	3 (3.3%)	0.336
Neutropenia (ANC < 500/mm^3^)	77 (39.9%)	30 (29.7%)	47 (51.1%)	0.002
Prolonged neutropenia (≥3 weeks)	25 (13.0%)	7 (6.9%)	18 (19.6%)	0.009
Breakthrough IYI	37 (19.2%)	22 (21.8%)	15 (16.3%)	0.334
Concomitant bacteremia, within ±3 days ^§^	63 (32.6%)	30 (29.7%)	33 (35.9%)	0.362
Serum lactate (>2 mmoL/L)	27 (14.0%)	4 (4.0%)	23 (25.0%)	<0.001
Vasopressor requirement related to IYI	70 (36.3%)	9 (8.9%)	61 (66.3%)	<0.001
Renal replacement therapy related to IYI	21 (10.9%)	7 (6.9%)	14 (15.2%)	0.065
Mechanical ventilation related to IYI	38 (19.7%)	7 (6.9%)	31 (33.7%)	<0.001
Treatment failures at 2 weeks from IYI diagnosis	91 (47.2%)	21 (20.8%)	70 (76.1%)	<0.001

Abbreviations: ANC, absolute neutrophil count; CRBSI, catheter-related bloodstream infection; HSCT, hematopoietic stem cell transplantation; IYI, invasive yeast infection. * Aplastic anemia (n = 9), hemophagocytic lymphohistiocytosis (n = 7), chronic myeloid leukemia (n = 4), chronic lymphocytic leukemia (n = 3), Waldenstrom macroglobulinemia (n = 3), hemolytic anemia (n = 2), immune thrombocytopenic purpura (n = 2), monoclonal gammopathy of undetermined significance (n = 2), autoimmune hemolytic anemia (n = 1), β-thalassemia (n = 1), Castleman disease (n = 1), chronic myelomonocytic leukemia (n = 1), histiocytic sarcoma (n = 1), myelofibrosis (n = 1); ^†^ Hepatosplenic candidiasis (n = 5), gastrointestinal tract (n = 5); ^‡^ Central nervous system (n = 4), musculoskeletal system (n = 2), pulmonary (n = 2), ruptured esophagus (n = 1), skin (n = 1); ^§^ Among 63 IYI episodes with concomitant bacteremia, 79 bacterial isolates were identified.

**Table 2:** Species distribution of concomitant bacteremia in invasive yeast infections.

Species	n (%) (Total 79 Isolates)
Gram-positives cocci	49 (62.0%)
* Enterococcus* spp.	31 (39.2%)
* Enterococcus faecium*	27 (34.2%)
* Enterococcus faecalis*	4 (5.1%)
*Staphylococcus* spp.	15 (19.0%)
Coagulase-negative *Staphylococcus* spp. *	12 (15.2%)
* Staphylococcus aureus*	3 (3.8%)
Viridans group *Streptococcus* spp. ^†^	2 (2.5%)
* Granulicatella adiacens*	1 (1.3%)
Gram-positive rods ^‡^	7 (8.9%)
Gram-negative rods	23 (29.1%)
*Klebsiella pneumoniae*	9 (11.4%)
*Escherichia coli*	5 (6.3%)
*Acinetobacter baumanii*	4 (5.1%)
Other Gram-negative rods ^§^	5 (6.3%)

* *S.* *epidermidis* (n = 7), *S. hominis* (n = 2), *S. capitis* (n = 1), *S. haemolyticus* (n = 1), *S. warneri* (n = 1); ^†^ *Streptococcus mitis*/*oralis* (n = 1), *Streptococcus parasanguinis* (n = 1); ^‡^ *Corynaebacterium striatum* (n = 2), *Bacillus sonorensis* (n = 1), *Clostridium innocuum* (n = 1), *Cutibacterium acnes* (n = 1), *Lactobacillus* spp. (n = 1), *Listeria monocytogenes* (n = 1); ^§^ *Enterobacter cloacae* (n = 2), *Stenotrophomonas maltophilia* (n = 2), *Acinetobacter ursingii* (n = 1).

## Data Availability

The datasets presented in this study are not available due to ethical and privacy restrictions imposed by the Seoul St. Mary’s Hospital Institutional Review Board.

## Appendix A. Survival Analysis for IYIs

**Table A1:** Univariate and Multivariate Cox regression analysis for 42-day overall case fatality.

	Univariate		Multivariate	
	HR (95% CI)	*p*	Adjusted HR (95 % CI)	*p*
Sex	1.482 (0.975–2.255)	0.066	1.445 (0.928–2.249)	0.103
Age > 65	2.022 (1.328–3.080)	0.001	2.168 (1.381–3.405)	0.001
Lymphoid origin	1.026 (0.658–1.600)	0.908		
Myeloid origin	0.797 (0.528–1.202)	0.280		
Active disease status	0.970 (0.619–1.519)	0.894		
Cytotoxic	0.976 (0.642–1.483)	0.909		
HSCT hx	0.844 (0.539–1.323)	0.460		
Steroid	1.771 (1.115–2.715)	0.009	1.704 (1.071–2.713)	0.025
Candidiasis	1.061 (0.514–2.192)	0.872		
CRBSI	0.554 (0.338–0.910)	0.020	1.285 (0.676–2.441)	0.444
Fungemia	1.815 (1.202–2.739)	0.005	1.695 (0.984–2.919)	0.057
Neutropenia	1.933 (1.283–2.913)	0.002	2.329 (1.467–3.697)	<0.001
Breakthrough IYI	0.732 (0.421–1.272)	0.268		
Concomitant bacteremia	1.299 (0.848–1.989)	0.229		
Elevated serum lactate level (>2 mmoL/L)	4.175 (2.583–6.750)	<0.001	4.077 (2.348–7.080)	<0.001
